# Probable endometrial tuberculosis in a patient with rhupus

**DOI:** 10.4102/sajid.v38i1.543

**Published:** 2023-10-26

**Authors:** Nimmisha Govind, Tamara Romanini, Lai Ling Winchow

**Affiliations:** 1 Department of Internal Medicine, Faculty of Health Sciences, University of the Witwatersrand, Johannesburg, South Africa; 2 Department of Internal Medicine, Chris Hani Baragwanath Academic Hospital, Johannesburg, South Africa

**Keywords:** endometrial, tuberculosis, rhupus, immunocompromised, rheumatoid arthritis, systemic lupus erythematosus

## Abstract

Endometrial tuberculosis (TB) is an uncommon manifestation of disseminated TB. Rhupus is the coexistence of rheumatoid arthritis (RA) and systemic lupus erythematosus (SLE). We describe a case of endometrial TB in rhupus patient an immunosuppressed.

**Contribution:**

We describe an uncommon presentation of disseminated TB, endometrial TB, in a rare rheumatic disease, rhupus. A high index of suspicion for TB is imperative in immunocompromised patients presenting with chronic urogenital symptoms especially in an endemic area.

## Introduction

There is a high prevalence of tuberculosis (TB) in rheumatic diseases driven by the immune dysregulation of the underlying disease, comorbidities and immunosuppressive therapy.^1^ Rhupus, a term used to describe the coexistence of rheumatoid arthritis (RA) and systemic lupus erythematosus (SLE), has a prevalence of 0.01% – 2.00% in patients with rheumatic conditions^2^ and is also associated with an increased risk of TB infection.^3^ We describe a case of endometrial TB in a patient with rhupus.

## Case

**FIGURE 1:**
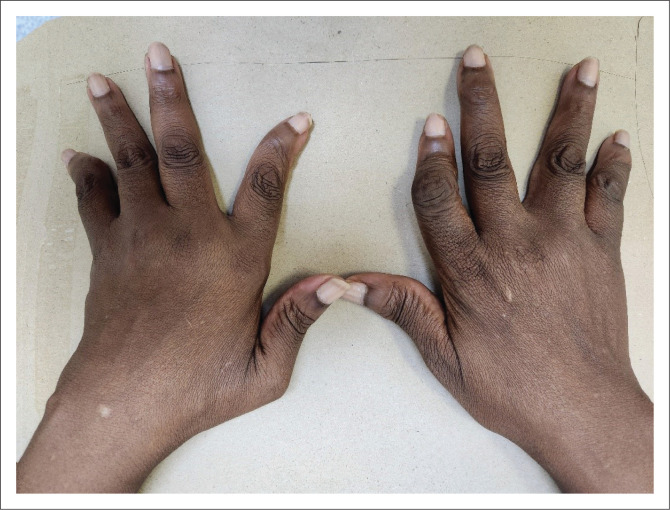
Deforming inflammatory arthritis.

A 57-year-old woman known with rhupus presented with a one-year history of non-offensive, ‘milky’ vaginal discharge, post-menopausal bleeding accompanied by loss of weight and appetite. Her diagnosis of rhupus was made in 2018 and was based on a deforming erosive inflammatory arthritis (Figure 1), painless oral ulcers, seizures, non-specific rashes and leucopenia with lymphopenia.

Investigations including anti-cyclic citrullinated peptide, anti-nuclear and anti-smith antibody were positive. Her oral treatment included methotrexate 10 mg weekly and chloroquine 200 mg from Monday to Friday. No glucocorticoids nor anti-tumour necrosis factor (TNF) agents were included in her treatment. Examination revealed that she was afebrile, with no lymphadenopathy or abdominal masses. Her rhupus was inactive. She was subsequently referred to gynaecology for a pap smear and further assessment.

**FIGURE 2:**
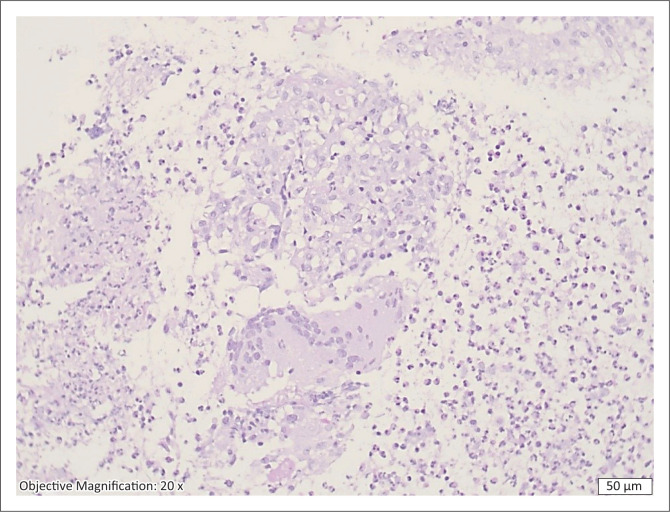
Necrotising granulomatous inflammation with a giant cell (haematoxylin- and eosin-stained section, × 200 magnification).

**FIGURE 3:**
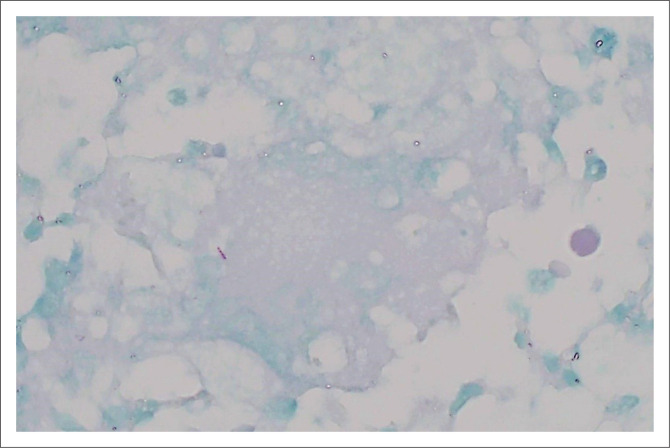
Acid-fast bacillus within a Langhans giant cells (modified Ziehl-Neelsen stain, oil emersion).

Her pap smear was normal, and a solid uterine mass was found on the pelvic ultrasound. During the admission for the biopsy of the uterine mass, she developed chest pain and echocardiography showed a small pericardial effusion with no features of tamponade. Blood investigations revealed a normocytic anaemia (haemoglobin = 9.6 g/dL) and mild thrombocytosis (437 × 10^9^/L). The C-reactive protein and erythrocyte sedimentation rate were elevated (45 mg/L and 25 mg/L), respectively. These resolved on anti-TB therapy with the C-reactive protein and erythrocyte sedimentation rate decreasing to 3 mg/L and 11 mm/h, respectively. The HIV enzyme-linked immunosorbent assay test was negative, and the chest radiograph was normal. Sputum PCR for *Mycobacterium tuberculosis*, Auramine O stain and TB culture were all negative. Microscopic examination of the uterine sections showed prominent necrotising granulomatous inflammation and suppurative exudate. There were scattered intermixed foreign-body and Langhans-type giant cells (Figure 2). The modified Ziehl-Neelsen stain was positive for acid-fast bacilli (Figure 3). The histopathology report supported active mycobacterial infection inducing necrotising granulomatous inflammation. Limitations of diagnostics include a lack of culture on biopsy as well as no CT scan nor MRI. She was initiated on anti-tuberculous treatment with resolution of her symptoms within 2 months. Tuberculosis diagnosis is supported by the above histology as well as a positive clinical and biochemical response to TB treatment.

## Discussion

Tuberculosis occurs in higher prevalence in SLE ranging from 5% to 30%.^4^ Rheumatoid arthritis patients have a four times higher risk of TB compared with the general population.^5^ A total of 20% of patients have a history of TB prior to SLE onset suggesting that TB may precipitate SLE. Extrapulmonary TB rates occur much higher in SLE patients occurring in two thirds of patients mainly the pleura, skin, lymph nodes, meninges and joints with a mortality of 31%.^6,7^ The risk of TB is significantly increased among RA and SLE patients with anti-TNF and glucocorticosteroid use.^5,6,8^ There is a paucity of studies on TB and rhupus.

Urogenital TB is a common extrapulmonary manifestation occurring in close to 30% of all extrapulmonary TB cases.^9^ The fallopian tubes (95% – 100%) are the most common female genital organ to be infected with TB followed by the endometrium (50% – 60%). Female genital TB may be asymptomatic or presents with infertility, changes in the menstrual cycle, pelvic pain and vaginal discharge. Importantly, endometrial TB may present like in our patient, with symptoms similar to that of endometrial cancer with continuous leucorrhoea, post-menopausal bleeding and pyometra in postmenopausal women.^10^ Physical examination may be normal (as in our patient) or may reveal an adnexal mass, adnexal tenderness, uterine irregularity or uterine prolapse.^11^ There should be a high index of suspicion in women with chronic pelvic inflammatory disease not responding to antibiotics, infertility and in postmenopausal bleeding, irregular menstrual cycles and persistent vaginal discharge.

Endometrial TB is usually spread haematogenously from pulmonary or extrapulmonary sites of TB, less commonly through descending direct and lymphatic spread.^12^ The bacilli can remain dormant in the tract and reactivate in immunosuppressive states. The patient most likely had primary pericardial TB, which spread to the endometrium; however, although probable, it is uncertain whether the pericardial effusion was definitely related to TB or possibly related to rhupus or another cause. The diagnosis of extrapulmonary TB can be made through the identification of one culture-positive specimen, positive histology or strong clinical evidence consistent with active extrapulmonary TB. A chest X-ray and ESR may be helpful. The two main imaging techniques useful in endometrial TB are hysterosalpingography and ultrasonography. Biopsy and bacteriological confirmation through acid-fast bacilli on Ziehl-Neelsen stain or isolation of *M. tuberculosis* on culture is necessary for the diagnosis. The World Health Organization, in its guidelines, recommended daily therapy of rifampicin, isoniazid, pyrazinamide and ethambutol for 2 months followed by daily 4-month therapy of rifampicin and isoniazid. In patients with persistent symptoms, surgical treatment should be considered and performed at least 6 weeks post anti-TB medication initiation to reduce the risk of post-operative complications.^10,13^

Endometrial TB and rheumatic diseases are rarely described with just one case previously described in a SLE patient on cyclophosphamide. The risk of TB in rhupus patients has not been established yet.

## Conclusion

We presented a rare case of a patient with rhupus on immunosuppressive therapy with a chronic vaginal discharge and a pelvic mass because of probable endometrial TB, with likely spread from the pericardium. A high index of suspicion for TB is imperative in immunocompromised patients presenting with chronic urogenital symptoms, especially in an endemic area. Histopathological assessment is important in the diagnosis of TB as all other investigations may be inconclusive. With early detection and appropriate management, urogenital TB has a good prognosis and low relapse rate.^14^ We therefore recommend thorough evaluation and investigation in patients presenting with symptoms suggestive of urogenital TB as well as early appropriate management.
